# Multisource feedback to assess pediatric practice: a systematic review

**Published:** 2013-03-31

**Authors:** Samah Al Alawi, Ahmed Al Ansari, Ayman Raees, Salman Al Khalifa

**Affiliations:** 1 Department of Family Medicine, Bahrain Defense Force Hospital, Riffa, Bahrain; 2 Department of General Surgery, Bahrain Defense Force Hospital, Riffa, Bahrain; 3 Department of Pediatrics, Bahrain Defense Force Hospital, Riffa Bahrain

## Abstract

**Introduction:**

The assessment and maintenance of competence for pediatricians has recently received increased attention. The aim of the present study was to investigate further the use of multisource feedback for assessing pediatricians in practice.

**Methods:**

A systematic literature review was conducted using the electronic databases EMBASE, PsycINFO, MEDLINE, PUBMED, and CINAHL for English-language articles.

**Results:**

762 articles were identified with the initial search and 756 articles were excluded for a total of six studies that met the inclusion criteria for this systematic review. Internal consistency reliability was reported in five studies with *α* ≥ 0.95 for both subscales and full scales. Generalizability was also reported in two studies with *Ep*^2^ generally ≥ 0.78. These adequate *Ep*^2^ coefficients were achieved with different numbers of raters. Evidence for content, criterion-related (e.g., Pearson’s *r*) and construct validity (e.g., principal component factor analysis) was reported in all 6 studies.

**Conclusion:**

Multisource feedback is a feasible, reliable, and valid method to assess pediatricians in practice. The results indicate that multisource feedback system can be used to assess key competencies such as communication skills, interpersonal skills, collegiality, and medical expertise. Further implementation of multisource feedback is desirable.

## Introduction

Challenges for pediatrics as a specialty started early in the 20^th^ century, when it was first accepted as a unique specialty – one that was defined and developed by physicians with the conviction that children and their illnesses require special attention and interest from staff who are highly skilled in their care. Pediatrics as a separate specialty has led to many advances in child health, including eradication of serious diseases such as rickets and scurvy. The establishment of this unique specialty also led to understanding the importance of high standards of child care and better medical education.^1^

In recent years, pediatricians continue to face challenges in identifying the best method to evaluate, and provide feedback to, their trainees in order to maintain high standards for graduating pediatricians. Physicians in general and pediatricians in particular have very little opportunity to receive systematic feedback about their practice. This is particularly the case for competencies like professionalism, communication skills, medical knowledge, and interpersonal relationships. It would, of course, be a matter of concern if underperformers, particularly pediatricians, were not detected. This problem can be addressed by introducing an assessment method to identify underperforming trainees and to help them in recognizing their problems and enhancing their performance.^2^

Multisource feedback (MSF) has emerged as a common method for assessing communication, professionalism, collaboration, and competence in the workplace.^3^ The feasibility, validity, and reliability of this assessment method was demonstrated by research in both industry and healthcare.^3^

The use of MSF has gained widespread acceptance and is seen as formative for reflecting on where change is required. Pediatricians complete a self-assessment instrument and receive feedback from medical colleagues (peers), co-workers (e.g., nurses, pharmacists), and patients (or patients’ parents or guardians).^4^,^5^ This feedback system using questionnaires by different personnel (the assessed person as well as colleagues, peers and clients) provides a more global perspective than can be provided by one or a few sources alone.^6^ Certain characteristics of health professionals such as clinical skills, personal communication, and patient or client management, combined with improved performance, can be assessed by MSF.

Multisource feedback is gaining acceptance and credibility as a method of providing pediatricians with the required information that helps them in monitoring and improving their performance and maintaining competence. Some studies of MSF have been conducted with pediatricians^7^ but there is not yet conclusive evidence about its effectiveness for assessing various competencies such as professionalism, communication skills, medical knowledge, clinical skills and interpersonal relationships.

The main purpose of the present study, therefore, was to conduct a systematic literature review to describe the use of MSF in pediatric settings and to determine its psychometric characteristics and evidence of its validity based on the published literature.

## Methods

The guidelines of the Preferred Reporting Items for Systematic (PRISM) reviews and meta-analysis were followed for this systematic review.^8^

### Information sources and search

A systematic literature search was conducted of English-language studies published from 1975 to October 2012 for the following databases: MEDLINE, EMBASE, CINAHL, PubMed, and PsychINFO. The reference lists of selected articles were searched as well for potential articles about MSF. The following terms were used in the search: multisource feedback, multisource feedback in pediatric settings, 360 degree evaluation, and 360 degree evaluation in pediatric settings.

### Study selection criteria

Studies were included if they met the following criteria: published in English, peer review journals, identified factors measured by the instruments, applied to pediatricians or pediatric practice, included information on at least one of feasibility, reliability, generalizability, and validity of the MSF measure used, and described the instrument design. We excluded studies in non-pediatric specialties such as surgery, family medicine, anesthesiology etc., provided only general application and guidelines for MSF without empirical data, reported only about the process of MSF, only reported changes in performance after feedback.

#### Data collection process

Each article in this study was evaluated by 2 authors (SA, AA) independently based on the title and abstract. Any disagreements between the two coders were solved by retrieving the full article and reviewed by a third coder (AR, SAL). Based on discussions among the four coders, we achieved 100% agreement on studies to be included.

**Figure 1:**
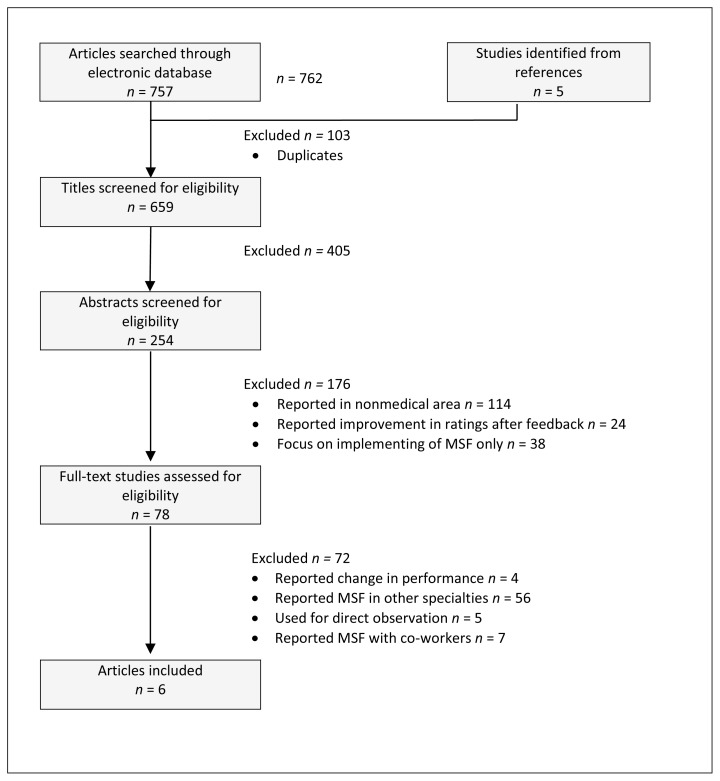
selection of studies for the systematic review

The initial search yielded 762 articles as described in Figure 1. Of these, 103 were duplicates, 405 articles were excluded based on the title, a further 176 articles were excluded based on the abstract and another 72 were eliminated after reading the full article. Finally we agreed on 6 articles to be included in the present study.

## Results

As summarized in Figure 1, of the 762 initial articles only 6 met the inclusion criteria and 756 were excluded. One study was published prior to 2005 (in 2004). The remaining five studies were published between the years of 2005 – 2010. Two studies were conducted in the USA, another two studies in the UK, and the last two studies in Canada (Table 1).

### Type of assessment instruments

Different instruments were used in the studies. Two studies used the Physician Achievement Review (PAR)^9^,^10^ instrument and another two used the Sheffield Patient Assessment Tool (SPRAT)^11^,^12^ to assess pediatricians. The remaining two studies used single questionnaires with variable numbers of items ranging from 10 to 14 across the instrument.^13^,^14^ The details of the studies are summarized in Tables 1 and 2. The instruments were designed to assess a range of competencies including communication skills, diagnostic and treatment skills, patient relationships, collegiality, leadership, decision making, system based practice, probity, professionalism, and knowledge and judgment (Table 1).

### Feasibility

In most of the studies, the response rates were more than 90%, which indicates the feasibility and acceptability of applying such assessment methods. Most of the studies used the response rate as an indication of feasibility. High response rates support the feasibility of the MSF process. Other papers demonstrated the feasibility of MSF by the time needed to complete the MSF forms (Table 2). Violato et al.^9^ reported high response rates for self (100%), medical colleague (95.5%), co-workers (94.8%), and patients (93.6%), across the PAR surveys. Lockyer et al.^10^ reported similar response rates (94.8%) for medical colleague using the PAR questionnaires. Other researchers identified the feasibility of the MSF by the time needed to complete MSF forms which generally took between six and fifteen minutes, depending on the number of items. Archer et al.^12^ reported that the mean time taken to complete the questionnaire by raters was six minutes. Feedback analysis and preparation of reports took an average of 30 minutes indicating that it is a feasible tool in real practice.

In several studies (especially those from Canada), participation in the MSF process is mandated by the regulatory or licensing authorities and, therefore, all pediatricians must participate to continue their medical practice (Table 2). In other studies,(e.g., in the UK and the US) MSF has been developed to assess pediatric residents and pediatricians by licensing authorities and by training programs. It appears feasible, therefore, to employ MSF for both trainees (e.g., residents) and practicing pediatricians.

### Reliability and generalizability

Reliability refers to the consistency of the measurement. Reliability coefficients are typically reported as Cronbach’s alpha (*α*) and reflect the internal consistency of the items. MSF instruments should have an *α* ≥ 0.90, which is typically achieved by most of the MSF instruments. Violato et al.^9^ reported reliability coefficients of *α* = 0.98, 0.98, 0.95, and 0.99, for self, medical colleague, co-worker, and patient instruments respectively.

Lockyer et al.^10^ reported a reliability coefficient of α = 0.98 for their 36-item medical colleague instrument. Similarly, Brinkman et al.^14^ reported reliability coefficients of *α* = 0.90, and 0.96 respectively for parents and co-workers questionnaires. Alternately, the calculation of a 95% CI for mean ratings by varying numbers of raters using generalizability theory is done to determine the number of raters needed to achieve a stable score, if the intent is to determine whether or not the person’s performance is satisfactory.^12^ In general, to achieve a standard error of measurement (SEM) ≤ 0.40 with the SPRAT instrument, a minimum of 8 raters is required.^15^ In the assessment of the SPRAT instrument for 577 pediatricians in training, Archer and associates determined that eight raters using a 24-item survey at a 95% CI provided ratings of a satisfactory level (SEM ≤ 0.40).^11^

Several researchers investigated the number of raters and the number of items required to provide stable data on the individual being assessed. This can achieved by employing generalizability theory to derive generalizability coefficients (*Ep*^2^).^15^ *Ep*^2^ provides a measure of the dependability of the MSF instruments as a function of the various factors that can influence the physicians’ ratings. Studies showed that it is possible to achieve adequate *Ep*^2^ ≥ 0.78 with a moderate number of observers.^11^ Generalizability was reported in only two studies and it was found that generalizability coefficients ranged from *Ep*^2^ = 0.78 to 0.87 with minimum of 8 peers and about 20 or more patients.^9^,^10^. Violato et al.^11^ achieved an *Ep*^2^ of 0.83 with 8 medical colleague raters. Lockyer et al.^10^ achieved an *Ep*^2^ = 0.78 for 8 medical colleagues, *Ep*^2^ = 0.85 with 8 co-workers, and *Ep*^2^ = 0.87 with 25 patients. The other four studies in Table 2 did not report generalizability analyses.

### Validity

Of the 6 studies included in the present systematic review (Table 1), only one reported evidence of content validity by determining if the content of the instrument was an adequate sample of the domain it was supposed to represent. Enhancing content validity of instruments (sampling of appropriate content and skills) can be achieved by using a table of specifications based on a list of core competency areas and methods to assess them and by having experts systematically review items to ensure that each competency is adequately assessed.^7^

Archer et al.^12^ reported the content validity for the SPRAT. Two authors wrote the questions, which were field tested in two pilot studies at the Sheffield Children’s Hospital. After modification following feedback, the final form contained 24 questions covering five domains, thus achieving content validity.

Criterion-related-validity was reported as well. Criterion validity refers to the relationship between scores obtained using the instrument and scores obtained using one or more other instruments or measures. Two studies (Table 1) supported criterion-related-validity (concurrent and predictive) by comparing the results of MSF scores across two different year levels.^11^,^12^ Archer et al.^11^ examined the predictive validity by comparing MSF scores between year two and year four trainees. The mean scores were calculated between year 4 trainees [*M* = 5.18, SD (0.34)] and year 2 trainees [*M* = 5.08, SD (0.34)] such that year 4 trainees scored significantly higher than year 2 (*p* < 0.01). In another study, Archer et al.^12^ examined the predictive validity by comparing MSF scores between senior house officers (SHO) and specialist registrar (SPRs) trainees. Specialist registrar trainees scored significantly higher than senior house officers, with a mean score ranging from SPRs [*M* = 5.22 (*SD* = 0.34) to SHO *M* = 4.81 (*SD* = 0.35), *p* < 0.001]. Consistently higher ratings given to advanced trainees by year of program support the criterion-related-validity of the MSF instruments.

Evidence for construct validity, which refers to the nature of the psychological construct or characteristic being measured by the instrument, was reported in all of the studies.^9^,^14^ Establishing construct validity can be achieved by studying the relationships among the latent variables or constructs. To do so, exploratory factor analysis can be used to determine the relationship among the variables. Violato et al.^9^ conducted a principal component factor analysis to derive a four factor solution for the medical colleague questionnaire accounting for 67.6% of the variance, a three-factor solution for the co-worker questionnaire, accounting for 63.8% of the variance, and a four-factor solution for the patient questionnaire, accounting for 77.6 % of the variance. Lockyer et al.^10^ also investigated the construct validity of the MSF instruments with very similar results.

In addition, the mean score was calculated between self-assessment and medical colleague assessment (MC). Pediatricians rated themselves lower than medical colleagues, with self *M* = 3.90 (*SD* = 0.76), and MC *M* = 4.45 (*SD* = 0.62). The mean score was also calculated between patient and (MC) assessments. Patients rated pediatricians higher than medical colleagues with patients, *M* = 4.63 (*SD* = 0.72), and MC *M* = 4.45 (*SD* = 0.62). This indicates that patients consistently rated trainees more leniently than other groups. These findings support construct validity.

Archer et al.^11^ conducted a principal component factor analysis to derive a two-factor solution accounting for 76.5% of the variance. They found that consultants rated trainees significantly lower (*t* = −4.52, *p* < 0.05), whereas senior house officers and foundation doctors [junior residents] scored their pediatric specialist registrars [senior residents] significantly higher (SHO *t* = 2.06, *p* < 0.05). This indicates that the consultants consistently rated trainees more stringently than other groups. These findings support construct validity.

Brinkman et al.^13^ examined the construct validity by comparing the mean score between a control group and an MSF group. The group that received feedback in the form of MSF scored higher than the control group. In addition, the mean score was calculated between time-one co-worker ratings *M* = 61 (*SD* = 5.25) and time-two co-worker ratings *M* = 68 (*SD* = 5.25) showing that ratings increased from time 1 to time 2.

Chandler et al.^14^ examined the construct validity in a different way. The mean score was calculated between self-assessment and assessment by medical colleagues. The mean ratings on the medical colleague instrument (approximately *M* = 4.85, *SD* = 0.32) are considerably higher than the self ratings (*M* = 4.44, *SD* = 0.43) by more than one standard deviation (*p* <.01). This is a typical finding as is found in much other MSF research where self ratings are below ratings by others.^9^

## Discussion

The main findings of the present study are : 1) MSF can be applied to pediatric practice both in residency and for licensing recertification; 2) MSF can assess various competencies such as diagnostic and treatment skills, patient relationships, collegiality, leadership, decision making, system based practice, probity, professionalism, knowledge and judgment, and communication; 3) different raters can be employed, such as medical colleagues, co-workers, supervisors, patients and self-assessment; 4) the MSF system is feasible with typically high response rates to questionnaires which require only a brief period of time to complete; 5) high internal consistency reliability of the instruments can be achieved; 6) as few as 8 raters and 23 patients can achieve an Ep^2^ coefficient ≥ 0.78, and 7). There is evidence of validity (content, criterion-related, construct) for the use of MSF in the assessment of pediatric practice.

A number of non-technical competencies such as leadership, decision making, system based practice, probity, professionalism, knowledge and judgment, and communication can effectively and feasibly be assessed using MSF for both pediatric trainees and independently practicing pediatricians. A full MSF model should include data from a self-assessment, medical colleagues (e.g., other pediatricians, referring physicians, anesthesiologists), co-workers (e.g., nurses, office staff), and patients (or patients’ relatives or parents).

Across the several studies reviewed, the internal consistency reliability reported is high and typically in excess of α = 0.98. Furthermore, the number of peer or co-worker raters required to assess a pediatrician is around 8. In particular, with well-designed MSF questionnaires in excess of about 17 items, the accepted standard for a generalizability coefficient of Ep^2^ ≥ 0.70 can be achieved. Nevertheless, approximately 25 patients are required to achieve a similar Ep^2^ coefficient.

Evidence for several sources of validity was examined. These include evidence of content, criterion-related and construct validity. Most of the construct validity evidence comes from factor analysis studies that identify the basis of constructs or domains (e.g., communication skills, professionalism, etc.) measured with the different MSF questionnaires. Future research may well include confirmative factor analyses which can provide stronger construct validity evidence.^16^

The present systematic review has some limitations. MSF assessments are entirely questionnaire-based and rely on judgment and inference by the assessors and respondents, which are known to be subject to a variety of influences and heuristics.^17^ Therefore, criterion-related validity studies of correlations between direct observations of behavior or performance and MSF scores are required to add further evidence of validity. MSF approaches fail to assess aspects of clinical competence reflecting pediatricians’ knowledge and skills; these may be more accurately obtained through other methods (e.g., chart reviews, traditional examinations). This systematic review is based on a relatively small number of studies (6) that were published in peer-reviewed, English-language journals. Further research should be done to replicate and extend some of the empirical findings, especially generalizability and validity evidence. Meanwhile the current empirical evidence is promising.

### Conclusion

This systematic literature review has shown that MSF is a feasible, reliable and valid method in assessing pediatricians in practice as well as pediatric trainees. The results indicate that multisource feedback systems can be used to assess key competencies such as communication skills, interpersonal skills, collegiality, and medical expertise. This feedback system can provide information to pediatricians for future professional development beyond that which can be provided by one or a few sources alone.^6^ Although reliability and validity challenges remain, MSF is a promising method for assessing pediatricians across a broad range of competences.

## Figures and Tables

**Table 1:** Description of the six studies on pediatricians multisource feedback included in the systematic analysis

Study name (Origin)	Specialty (*n*)	MSF Instrument Personnel (No. Items)	Constructs/Factors assessed	Validity

Violato et al. 2006^9^ (Canada)	Pediatrics (*n* = 100 pediatrician)	***PAR***		***Construct:*** Principal component factor analysis was conducted to derive a four factor solution for (MC) accounting for 67.6% of the variance, three factors for (CW) accounting for 63.8%, and four factor for (Pt) accounting for 77.6 %. Self-instrument is identical to co-worker instrument. ***Construct:*** The mean score was calculated between self-assessment and (MC). Pediatrician rated themselves lower than (MC) with self *M* = 3.90 (*SD* =0.76), and MC *M* = 4.45 (*SD* = 0.62). ***Construct:*** The mean score was calculated between Patients and (MC) assessments. Patients rated physician higher than MC with Pt, *M* = 4.63 (*SD* = 0.72), and MC *M* = 4.45 (*SD* =0.62).
		Self, (37 Items)	Prof, Clin comp, Comm	
		MC, (38 Items)	Prof, Clin comp, Comm	
		CW, (22 Items)	Comm, InterPer	
		Pt, (40 Items)	Prof, Comm, Mnger	

Lockyer et al. 2004^10^ (Canada)	Pediatrics (*n* = 100 pediatrician)	***PAR***		***Construct:*** Principal component factor analysis was conducted to derive a four factors for pediatric questionnaire accounting for 67.6% of the variance.
		MC (36 Items)	Prof, Clin comp, Comm	

Archer et al. 2010^11^ (UK)	Pediatrics (*n* = 577 residents)	***SPRAT***		***Construct:*** Principal component factor analysis was conducted to derive a two factor solution accounting for 76.5% of the variance. ***Construct:*** Consultants marked trainees significantly lower (*t* = −4.52, *p* < 0.05), whereas SHOs and foundation doctors scored their SPRs significantly higher (SHO *t* = 2.06, *p*< 0.05. ***Predictive:*** The mean scores were calculated between Year 4 trainees *M* = 5.18 (*SD* = 0.34) to Year 2 trainees *M* = 5.08 (*SD* = 0.34), *p* < 0.01. Year 4 scored significantly higher than year 2.
		MC, CW (24 Items)	Clin Comp, Inter Per	

Archer et al. 2005^12^ (UK)	Pediatrics (*n* = 112 residents)	***SPRAT***		***Construct:*** The mean scores were calculated between Specialists registrar trainees *M* = 5.22 (*SD* = 0.34) to senior house officers *M* = 4.81 (*SD* = 0.35), [*t* = − 4.765, *df* =110, *p* < 0.001].Specialist’s registrar trainees scored significantly higher than senior house officers since they are senior to them.
		MC, CW (24 items)	Clin Comp, Inter Per	

Brinkman et al. 2007^13^ (USA)	Pediatric (*n* = 36 residents)	***Multisource feedback***		***Construct:*** The Mean score was calculated for control group and MSF group. MSF group scored higher than control group with *M* = 68 (*SD* = 5.2) vs. *M* = 50 (*SD* = 7.0), respectively. ***Predictive:*** The mean scores were calculated between Time 1 CW ratings *M* = 61 (*SD* = 5.25) to Time 2 CW *M* = 68 (*SD*= 5.25), CW ratings increased from Time 1 to Time 2.
		Parents (10 Items)	Prof, Comm	
		CW (14 Item)	Prof, Clin Comp, Comm	

Chandler et al. 2010^14^ (USA)	Pediatrics (*n* = 66 residents)	**360 degree evaluation**		***Construct:*** The mean score was calculated between self-assessment and (MC). Pediatric residents rated themselves lower than (MC) with self *M* = 4.44 (*SD* = 0.43), *p* < 0.001.
		Self(10 Items)	Prof, Comm	
		MC (10 items)	Prof, Comm	
		CW (10 Items)	Prof, Comm	
		Pt (10 Items)	Prof, Comm	

PAR = Physician Achievement Review, Prof = Professionalism, Clin Comp = clinical competence, InterPer = Interpersonal Relationship, Comm = Communication, MC = Medical colleague, CW = Co-Worker, Pt =Patient, Mngr = manager SPRAT = Sheffield Peer Review assessment Tool

SHOs = Senior House Officer, SPRs =Pediatric Specialists Registrar, MSF = Multi Source Feedback, SEM = Standard Error of Measurement.

*Professionalism* covers: Psychosocial skills, psychosocial management, Humanistic qualities, compassion, attitude, professional development, teaching, and professional responsibilities and professional management.

*Clinical competence* covers: Clinical care, good medical practice, patient care, safe practice, clinical performance, Knowledge, critical thinking, diagnosis, and management of complex problem.

*Communication* covers: Communication with staff, and interpersonal communication skills,

*Manager* covers: Reporting, self-management, administrative skills, office personnel, access to doctor, practice process, physical office, and physical space.

*Interpersonal relationship* covers: Relationship with patients, with colleague, with family member, collegiality, collaborator, patient education, information provision, and patients interaction), and the last factor is overall assessment.

Two of the authors (SA), and (AA) agreed on the names of the 6 main domains and the items included in each.

**Table 2:** Reliability and validity characteristics of the six studies on pediatricians’ multisource feedback

Study Name	Mean No. of Raters (% Response)	Reliability Coefficient (α) or [95% CI]	Administration/Feasibility	Generalizability (*Ep^2^*) or Intra-Class Correlation (ICC)

Violato et al. 2006^9^ (Canada)	Self, 1 (100%)	self, α = 0.98	The college of physicians and surgeons of Alberta (CPSA) introduced the PAR instruments to evaluate pediatricians in clinical practice. The aim of developing those surveys was to extend the use of PAR instrument to the evaluation of pediatricians and to assess the feasibility, reliability, and validity of MSF system in pediatric practice setting.	
	MC, 7.64 (95.5%)	MC, α = 0.98		7.64 MC, Ep^2^= 0.78
	CW, 7.58 (94.8%)	CW, α = 0.95		7.58 CW, *Ep^2^*= 0.87
	Pt, 23.41 (93.6%)	Pt, α = 0.99		23.41 Pt, *Ep^2^*= 0.85

Lockyer et al. 2004^10^ (Canada)	MC, 7.6 (94.8%)	MC, α = 0.98	This instrument was implemented to determine whether a common peer assessment instruments can provide a valid and reliable assessment of competencies across different specialties. The authors concluded that single instrument is appropriate for use across different specialties such as pediatric, internal medicine, and psychiatrists.	7.6 MC, *Ep^2^* = 0.83

Archer et al. 2010^11^ (UK)	MC, CW 8.26 (83%)	SEM for 8 raters ± 0.40 (95% CI)	SPRAT was developed to assess the generic competencies of Good Medical Practice (GMP) as a national implementation mandate with the use of the MSF for Pediatric Specialist Registrars (SPRs).	NR
Archer et al. 2005^12^ (UK)	Combined MC and CW 8.2 (82%)	SEM for 4 raters ± 0.50 (95% CI)	The authors concluded that, the use of the Sheffield Peer Review Assessment Tool (SPRAT) was a feasible, reliable and valid assessment method in informing the record of in-training assessment for pediatric senior house officers and specialists’ registrars. The feedback from SPRAT can also be used to inform personal development planning and focus quality improvement.	NR

Brinkman et al. 2007^13^ (USA)	Parents, 19.25	Parents, α = 0.95	This instrument was introduced to determine whether augmentation standard feedback on resident performance with a multisource feedback intervention improved pediatric resident communication skills and professionalism. These questionnaires were shown to enhance standard feedback on resident performance and improved their communication skills and professionalism.	
	CW, 15,8	CW, α = 0.96		NR

Chandler et al. 2010^14^ (USA)	Self, 1 MC, 2.6 CW, 7.4 Pt, 1.2	NR	The aim of this study was to determine if non-faculty ratings of resident’s professionalism and interpersonal skills differ from faculty rating. Overall, the 360 degree evaluation ratings for the pediatric residents were high and provided guidance to them their interpersonal and communication skills.	NR

* NR = not reported

## References

[1] Zipursky A (2002). A history of pediatric specialties. Pediatric Research.

[2] Lockyer J, Clyman S, Holmboe ES, Hawkins RE (2008). Multisource feedback (360-degree Evaluation). Practical guide to the evaluation of clinical competence.

[3] Wilson RM, Harrison BT, Gibberd RW, Hamilton JW (1999). An analysis of adverse events from the quality in Australian health care study. Med J Austral.

[4] Sala F, Dwight S (2002). Predicting executive performance with multi-rater surveys: Whom you ask makes a difference. Consult Psychol J.

[5] Violato C, Lockyer J, Fidler H (2007). Assessment of psychiatrists with multisource feedback. Can J Psychol.

[6] Bracken DW, Church AH (2001). The handbook of multisource feedback: *The comprehensive resource for designing and implementing MSF processes*.

[7] Violato C, Lockyer J, Fidler H (2003). Multisource feedback: a method of assessing surgical practice. Brit Med J.

[8] Moher D, Liberati A, Tetzlaff J, Altman DG (2009). Preferred reporting items for systematic reviews and meta-analysis: the PRISMA statement. Brit Med J.

[9] Violato C, Lockyer J, Fidler H (2006). Assessment of pediatricians by a regulatory authority. Pediatrics.

[10] Lockyer J, Violato C (2004). An examination of the appropriateness of using a common peer assessment instrument to assess physician skills across specialties. Acad Med.

[11] Archer J, McGraw M, Davies H (2010). Assuring validity of multisource feedback in national program. Arch Dis Childhood.

[12] Archer J, Norcini J, Davies A (2005). Use of SPRAT for peer review of paediatricians in training. Brit Med J.

[13] Brinkman WB, Geraghty SR, Lanpher BP, Khaury J, Gonzalez J, Dewitt T, Britto M (2007). Effect of multisource feedback on resident Communication skills and professionalism. Arch Ped Adoles Med.

[14] Chandler N, Henderson G, Park B, Byerley J, Brown W, Steiner M (2010). Use of a 360-Degree evaluation in the outpatient setting: The usefulness of Nurse, Faculty, Patient/Family, and Resident self-evaluation. J Grad Med Educ.

[15] Archer J, Norcini J, Southgate L, Heard S, Davies H (2008). Mini-PAT (Peer Assessment Tool): A valid component of a national assessment program in the UK?. Adv Health Sci Educ.

[16] Violato C, Hecker K (2007). How to use structural equation modeling in medical education research: A brief guide. Teach Learn in Med.

[17] Kahneman D (2011). Thinking, fast and slow.

